# OpenSAFELY: impact of national guidance on switching anticoagulant therapy during COVID-19 pandemic

**DOI:** 10.1136/openhrt-2021-001784

**Published:** 2021-11-16

**Authors:** The OpenSAFELY Collaborative, Helen J Curtis, Brian MacKenna, Alex J Walker, Richard Croker, Amir Mehrkar, Caroline Morton, Seb Bacon, George Hickman, Peter Inglesby, Chris Bates, David Evans, Tom Ward, Jonathan Cockburn, Simon Davy, Krishnan Bhaskaran, Anna Schultze, Christopher T Rentsch, Elizabeth Williamson, William Hulme, Laurie Tomlinson, Rohini Mathur, Henry Drysdale, Rosalind M Eggo, Angel Yun Wong, Harriet Forbes, John Parry, Frank Hester, Sam Harper, Ian Douglas, Liam Smeeth, Ben Goldacre

**Affiliations:** 1 The DataLab, Nuffield Department of Primary Care Health Sciences, University of Oxford, Oxford, UK; 2 TPP, Leeds, UK; 3 Department of Non-communicable Disease Epidemiology, London School of Hygiene & Tropical Medicine, London, UK; 4 Department of Medical Statistics, London School of Hygiene & Tropical Medicine, London, UK; 5 Department of Infectious Disease Epidemiology, London School of Hygiene & Tropical Medicine, London, UK; 6 Population Health Sciences, Bristol Medical School, University of Bristol, Bristol, UK

**Keywords:** COVID-19, healthcare economics and organisations, medication adherence, stroke

## Abstract

**Background:**

Early in the COVID-19 pandemic, the National Health Service (NHS) recommended that appropriate patients anticoagulated with warfarin should be switched to direct-acting oral anticoagulants (DOACs), requiring less frequent blood testing. Subsequently, a national safety alert was issued regarding patients being inappropriately coprescribed two anticoagulants following a medication change and associated monitoring.

**Objective:**

To describe which people were switched from warfarin to DOACs; identify potentially unsafe coprescribing of anticoagulants; and assess whether abnormal clotting results have become more frequent during the pandemic.

**Methods:**

With the approval of NHS England, we conducted a cohort study using routine clinical data from 24 million NHS patients in England.

**Results:**

20 000 of 164 000 warfarin patients (12.2%) switched to DOACs between March and May 2020, most commonly to edoxaban and apixaban. Factors associated with switching included: older age, recent renal function test, higher number of recent INR tests recorded, atrial fibrillation diagnosis and care home residency. There was a sharp rise in coprescribing of warfarin and DOACs from typically 50–100 per month to 246 in April 2020, 0.06% of all people receiving a DOAC or warfarin. International normalised ratio (INR) testing fell by 14% to 506.8 patients tested per 1000 warfarin patients each month. We observed a very small increase in elevated INRs (n=470) during April compared with January (n=420).

**Conclusions:**

Increased switching of anticoagulants from warfarin to DOACs was observed at the outset of the COVID-19 pandemic in England following national guidance. There was a small but substantial number of people coprescribed warfarin and DOACs during this period. Despite a national safety alert on the issue, a widespread rise in elevated INR test results was not found. Primary care has responded rapidly to changes in patient care during the COVID-19 pandemic.

**Key questions**

**What is already known about this subject?**

- Since the beginning of the COVID-19 pandemic, the National Health Service (NHS) has recommended putting measures in place to reduce face-to-face contact with patients where possible to reduce the risk of transmission. This includes guidance to consider prescribing a direct-acting oral anticoagulant (DOAC) instead of warfarin in suitable patients. However, it was not known how widespread this practice was, or whether it results in potentially dangerous coadministration. In addition, there has one small report on reduced international normalised ratio (INR) control during the pandemic.

**What does this study add?**

- This is the first widespread study on the impact of the NHS England guidance. It shows that a substantial number of patients in general practice in England had their anticoagulant switched from warfarin to a DOAC. It also shows that there was a small number of patients who potentially received both warfarin and a DOAC. We also showed that there did not appear to be a widespread reduction in INR control during the pandemic.

**How might this impact on clinical practice?**

- This study highlights that national prescribing guidance can be implemented in primary care in England, both on a large scale and at a fast pace when necessary to improve patient care. It also highlights the importance of good clinical governance processes when switching medications, in order to reduce the risk of coprescribing of both agents.

## Background

Anticoagulants are prescribed to people at risk of or for treatment of thromboembolism, which in some cases can result in stroke. Warfarin, a vitamin K antagonist, has been the mainstay of oral anticoagulant treatment for decades. A patient’s specific dose of warfarin is adjusted based on frequent blood tests that determine their international normalised ratio (INR), a measurement of blood clotting time. Factors such as changes in diet, alcohol intake, acute illness and concomitant medications can affect blood levels of warfarin and INR, requiring a temporary increase in frequency of testing. The quality of the anticoagulation control is assessed by the proportion of time in therapeutic range (TTR). Direct-acting oral anticoagulants’ (DOACs; rivaroxaban, dabigatran etexilate, apixaban and edoxaban) mechanism of action does not alter the INR and therefore people taking DOACs require less frequent drug safety monitoring (eg, renal function). Nationally, the prescribing of DOACs has increased steadily since their recommendation by NICE for atrial fibrillation (AF) in 2012.^1^

Following the onset of the COVID-19 pandemic, the National Health Service (NHS) responded to deliver healthcare services in a manner that minimised risk of virus transmission. Most patients taking the anticoagulant warfarin require frequent blood tests, the INR test, potentially increasing their chance of exposure to SARS-CoV-2. NHS England issued guidance in March 2020^2^ to support local NHS organisations to manage their anticoagulant services; this included identifying people suitable for switching from warfarin to DOACs. In May 2020, NHS England wrote to Clinical Commissioning Groups (CCGs), the local NHS bodies responsible for medicines use, advising that apixaban or rivaroxaban should be prescribed in place of warfarin for people able to change,^3^ following a procurement exercise that secured additional stock at reduced prices. In October 2020, the Medicines and Healthcare products Regulatory Authority (MHRA) issued a safety alert, warning about an increase in the number of people with substantially elevated INR levels observed during the pandemic and also warned that of some people for whom warfarin was inadvertently continued after switching to DOACs^4^; however, this document gave no indication of the scale of these problems.

Using a retrospective cohort study design, we set out to: evaluate the proportion and characteristics of prior warfarin users who switched to DOACs and how many subsequently reverted; identify potentially unsafe coprescribing of warfarin and DOACs; and measure the frequency of INR testing during the pandemic for people taking warfarin, any changes to TTR and the extent to which elevated INRs were observed. This was conducted as a ‘proof of concept’ for the use of the new OpenSAFELY analytics platform to rapidly understand service impacts during the COVID-19 pandemic and inform support for primary care.

## Methods

### Study design

Prescribing and testing practice was analysed by conducting a retrospective cohort study using data from English NHS general practices.

### Data sources

Primary care records managed by the General Practice (GP) software provider TPP were assessed using OpenSAFELY, a data analytics platform created by our team on behalf of NHS England to address urgent COVID-19 research questions (https://opensafely.org). OpenSAFELY provides a secure software interface allowing the analysis of pseudonymised primary care patient records from England in near real-time within the Electronic Health Record (EHR) vendor’s highly secure data centre, avoiding the need for large volumes of potentially disclosive pseudonymised patient data to be transferred off-site. This, in addition to other technical and organisational controls, minimises any risk of reidentification. Similarly, pseudonymised datasets from other data providers are securely provided to the EHR vendor and linked to the primary care data. The dataset analysed within OpenSAFELY is based on 24 million people currently registered with GP surgeries using TPP SystmOne software (40% of England’s population). It includes pseudonymised data such as coded diagnoses, medications and physiological parameters but no free-text data. Further details on our information governance can be found under ethics approval.

### Data processing

We extracted data for all people who were issued with a prescription or had an active repeat prescription for warfarin or DOACs between January 2019 and August 2020, and the relevant dates of each prescription and INR test-related activity. We used warfarin, DOAC and INR codelists from OpenSAFELY^5 6^ to identify these activities, with ‘high INRs’ classified as INR of >8/≥8 (as indicated) in line with thresholds relied on by the MHRA.^7^ We identified TTR using the following CTV3 codes ‘YavzQ’, ‘42QE2’ (redundant) and ‘Xaa68’.

### People switching from warfarin to DOACs following NHS England guidance

**Figure 1:**
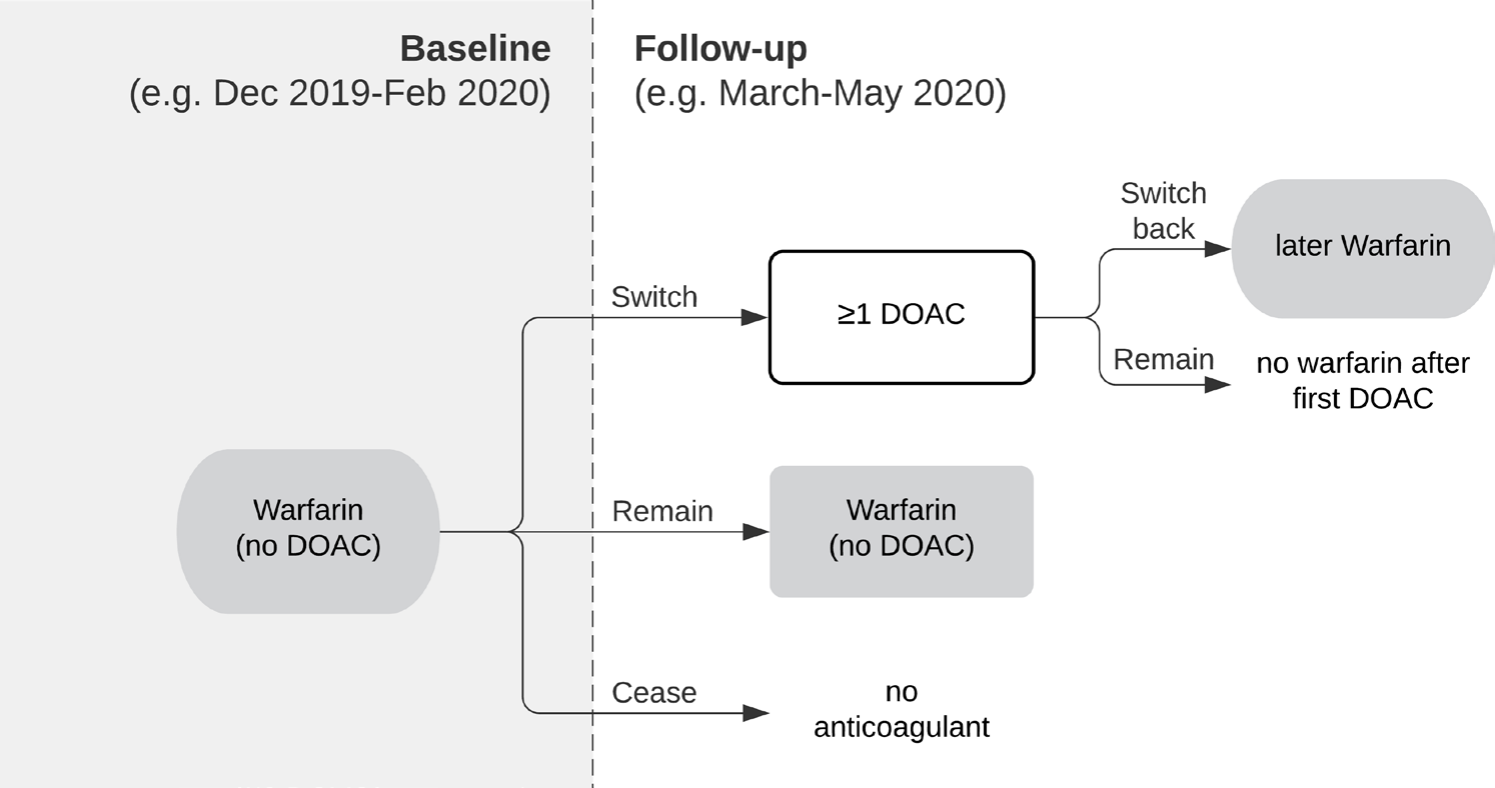
Identification of patients. Flow chart illustrating how patients switching from warfarin to DOACs (and those switching back within the same period) were identified based on prescriptions issued. DOACs, direct-acting oral anticoagulants.

We identified people issued a prescription for warfarin (but no DOACs) between December 2019 and February 2020 and assessed how many received at least one DOAC (‘switched’) in March–May 2020 or only received warfarin (figure 1). Of those switching, we counted how many later received warfarin again during this period (‘switched back’). Of those remaining on warfarin, we counted how many received an INR test, a high INR value or had a TTR recorded. This analysis was repeated for the subsequent 3-month period (warfarin March–May 2020, switching June–August 2020) and for the corresponding periods in the previous year. Descriptive tables were generated to describe the cohort.

We also assessed the overall rate of people starting new DOAC repeat prescriptions each month from January 2019 to August 2020 (with ‘new’ defined as people having no DOAC repeat prescription ending within the previous 3 months). We assessed how many of whom had switched from warfarin, defined as people having a warfarin repeat prescription with an end date in the previous 3 months. Figures were plotted on a time trend chart.

### Potentially unsafe coprescribing of warfarin and a DOAC

For each month, January 2019–August 2020, patients were identified who: (A) were issued a prescription for DOAC and warfarin on the same day or (B) had a repeat medication initiated for both DOAC and warfarin on the same day (and also had at least one warfarin/DOAC prescription issued within the month). A repeat prescription allows items to be issued, therefore having active repeats for both medications could allow both warfarin and a DOAC to be issued to a patient in error (however, prescriptions can also be issued acutely without a repeat). We identified whether these people had at least one of these cancelled the same day. Figures were plotted on a time trend chart.

### INR tests during the COVID-19 period

Each month, INR tests and TTR values were identified for people on warfarin (defined as people with warfarin issued within the previous 3 months, and no DOAC issued after the latest warfarin). We plotted the rate per 1000 warfarin patients (and for TTRs, also the rate per 1000 INR tests and the rate per 1000 people tested) on time trend charts.

### Factors associated with switching from warfarin to a DOAC during the pandemic

We investigated factors associated with switching from warfarin to DOAC using mixed effects logistic regression. We included the variables in table 1 as factors in the model. We excluded ethnicity due to ~30% missingness. We also planned to include the patient’s practice, but this was later removed to allow the model to converge. The population was: people aged 18–110 years at the start of the follow-up period; registered with a single practice for the 6 months prior to the follow-up period (to ensure completeness of covariate data); prescribed warfarin at least once in the baseline period; first prescribed warfarin at least 6 months ago (to exclude very recent initiations); not prescribed a DOAC during the baseline period; and prescribed at least one DOAC or warfarin during the follow-up period (to exclude people who ceased treatment or died before receiving any prescription in this period). The outcome was the prescription of at least one DOAC during the follow-up period. Baseline period: 3 months prior to the national ‘stay at home’ order (16 December 2019–15 March 2020); follow-up period: the following 3 months (16 March 2020–15 June 2020).

**Table 1:** Variables used to assess factors associated with switching (present on the first day of the follow-up period unless otherwise stated)

Factor	Detailed information on coding	Codelist (where applicable)
Age group	<65, 65–74 and 75+	
Indices of Multiple Deprivation (IMD)	Deciles based on residential postcode	Supplied by TPP.
Sustainability and transformation partnership (STP) membership	STP of current registered practice – modelled as a random effect	STP are NHS administrative regions made up of one or more CCGs. Linkage to national STPs carried out by TPP.
Care/nursing home residence status	Yes/no	Classification supplied by TPP based on patient’s residential address at 1 February 2020 linked to CQC care home register.
Diagnosis of AF	Yes/no; any time prior to follow-up period	https://codelists.opensafely.org/codelist/opensafely/atrial-fibrillation /2020-07-09/
Renal function	Latest estimated Glomerular Filtration Rate (eGFR function) (<30, 30–59 and ≥60) in the 12 months to the end of the baseline period (or ‘no evidence’ for those with no creatinine recorded in this period).	eGFR calculated from creatinine records. Creatinine clearance is recommended renal function assessment for tailoring DOAC doses for individual people. Automated calculation of Creatinine Clearance (CrCl) is not yet available in OpenSAFELY, and eGFR is used as pragmatic alternative.
Recent renal function test	Yes/no; within last 4 months and up to the end of the study period (this conservatively allows tests slightly outside of the recommended 3-month period, and any time during the switching period).	Creatinine test or: ‘451’. Renal function test. ‘XacUK’ eGFR using creatinine (calculated using Chronic Kidney Disease-Epidemiology Collaboration (CKD-EPI) per 1.73 square metres.
Number of INR tests	0, 1–3, 4–6 or 7+ tests during the baseline period	https://codelists.opensafely.org/codelist/opensafely/international-normalised-ratio -inr/2020-10-22
Length of time on warfarin*	<2 years (since 16 March 2018). 2–≤6 years (since June 2014, date of latest National Institute of Health and Care Excellence (NICE) AF guidance).^22^ 6–<8 years (since March 2012, date of first NICE Technology Appraisal for a DOAC for AF).^23^ ≥8 years.	https://codelists.opensafely.org/codelist/opensafely/warfarin/2020-10-05/
Previously prescribed DOACs*	Yes/no; any time prior to the baseline period.	https://codelists.opensafely.org/codelist/opensafely/direct-acting-oral-anticoagulants -doac/2020-10-05/
Explicitly recorded contraindicaton to DOAC	Yes/no; any time prior to the follow-up period.	https://codelists.opensafely.org/codelist/opensafely/explicit-contraindication-to-doacs-direct-acting-anticoagulants/2020-10-19/

*The earliest actual date of an event may be missing due to incomplete patient records (eg, lost in transfer between practices), but the earliest recorded date can be used as an approximation.

AF, atrial fibrillation; CCGs, Clinical Commissioning Groups; DOAC, direct-acting oral anticoagulant; INR, international normalised ratio; NHS, National Health Service.

### Software and reproducibility

Data management was performed using Python V.3.8 and SQL, and regression analysis using Stata V.16.1. All codes for the OpenSAFELY platform, and for data management and analyses for this study, are available for inspection and reuse under open licenses on GitHub (https://github.com/opensafely/anticoagulant-switching-research). All codelists are available for inspection and reuse from https://codelists.opensafely.org.

### Patient and public involvement

Patients were not formally involved in developing this specific study design that was developed rapidly in the context of a global health emergency. We have developed a publicly available website https://opensafely.org/ through which we invite any patient or member of the public to contact us regarding this study or the broader OpenSAFELY project.

## Results

### Patients switching from warfarin to DOACs following NHS England advice

A total of 164 000 people were prescribed warfarin between December 2019 and February 2020, of whom 12.2% (20 000) were prescribed a DOAC between March and May 2020 (table 2). This was substantially higher than the previous year (3.5%) and the following 3 months (4.4%) (table 2). Of those who switched to a DOAC, 5.8% (n=1200) also received a subsequent prescription for warfarin (switched back), compared with 4.1% the previous year (table 2). Of those remaining on warfarin, 80.1% had at least one INR test recorded during March–May compared with 83.7% the previous year, 38.6% had at least one recorded INR TTR (37.9% the previous year), and 0.5% had a high INR (≥8) result (0.4% the previous year; table 2). During March–May 2020, edoxaban and apixaban were the most commonly selected DOACs among those who were switched (38.1% and 33.5%, respectively; table 3). During June–August 2020, apixaban had increased to 40.3%, with edoxaban dropping to 33.4% (table 3).

**Table 2:** Warfarin patients switching to DOACs or remaining on warfarin

	Patient count, thousands (percentage)			
Period	March–May		June–August	
Year	2020	2019	2020	2019
Baseline warfarin patients**	164 000	195 000	143 200	185 900
Switched	20 000 (12.2)	6900 (3.5)	6300 (4.4)	5900 (3.2)
Continued warfarin	136 100 (83.0)	177 300 (90.9)	128 200 (89.5)	169 900 (91.4)
No anticoagulants†	7900 (4.8)	10 800 (5.6)	8700 (6.1)	10 100 (5.4)
Switched back (% of switchers)	1200 (5.8)	300 (4.1)	300 (4.7)	300 (4.4)
At least one INR (% of continued)	109 100 (80.1)	148 400 (83.7)	101 100 (78.8)	140 000 (82.4)
At least one TTR (% of continued)	52 600 (38.6)	67 200 (37.9)	50 300 (39.2)	66 500 (39.1)
High INR (≥8) (% of continued)	700 (0.5)	700 (0.4)	400 (0.3)	600 (0.3)

Number and percentage of warfarin patients who continued on warfarin, received a DOAC (‘switched’) or had no anticoagulants, during March–May 2020 compared with 2019 and similarly for June–August. Also showing the percentage of those switching to a DOAC who later received warfarin ‘switched back’ (within the same 3-month period); and the percentage of those remaining on warfarin who had at least one INR test, INR TTR or high INR (≥8) recorded. Patient counts are rounded to the nearest 100.

*Baseline warfarin patients for each period were those issued warfarin at least once (but no DOACs) in the 3 months immediately prior to the period shown.

†Those with no anticoagulants (referring to warfarin and DOACs only) in the follow-up period may have discontinued anticoagulant treatment, moved to a non-TPP practice, died or simply had a long period without a new prescription from their GP (eg, due to a hospital stay or longer than usual prescription duration).

DOACs, direct-acting oral anticoagulants; INR, international normalised ratio; TTR, time in therapeutic range.

**Table 3:** Types of DOAC selected

Period	Year	Apixaban		Edoxaban		Rivaroxaban		Dabigatran etexilate		Total
		Patient count	%	Patient count	%	Patient count	%	Patient count	%	
March–May	2020	6700	33.5	7620	38.1	5550	27.8	120	0.6	19 990
	2019	3510	51.1	1040	15.2	2190	31.9	120	1.8	6860
June–August	2020	2550	40.3	2110	33.4	1620	25.6	40	0.6	6320
	2019	2900	49.5	1110	18.9	1760	30	90	1.6	5860

Number and percentage of warfarin patients switched to each of the four types of DOAC between March and May 2020 compared with 2019, and similarly for June–August, for people who were prescribed warfarin during the previous 3-month period. Patient counts are rounded to the nearest 10. Percentages may not add to exactly 100 due to rounding.

DOAC, direct-acting oral anticoagulant.

**Figure 2:**
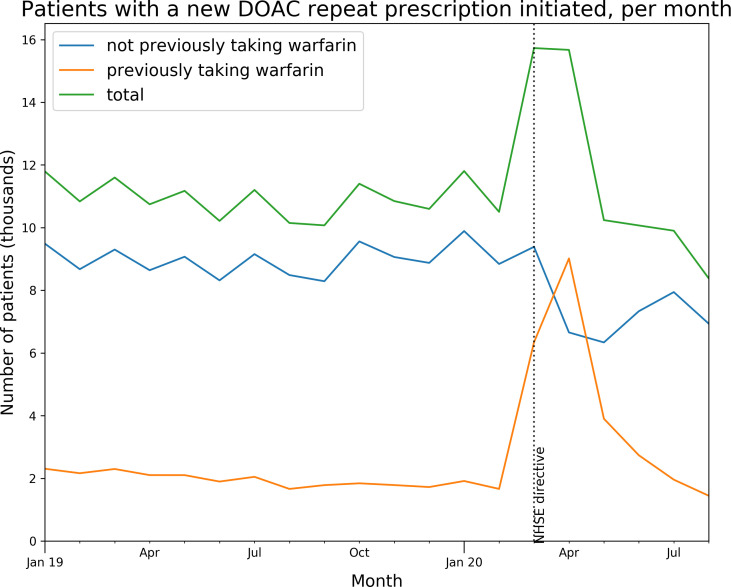
Patients newly initiated on DOAC repeats. Number of people having a new DOAC repeat prescription initiated per month (where the patient had no prior DOAC repeat ending within the previous 3 months), indicating whether or not patients had previously had a repeat prescription for warfarin (ending same month or within previous 3 months). DOAC, direct-acting oral anticoagulants.

The initiation of repeat prescriptions for DOACs to new patients increased ~1.5-fold during March and April 2020 and subsequently dropped slightly below normal levels (figure 2). Much of this increase was attributable to people switching from warfarin (40.3% in March 57.5% in April), compared with the normal rate of ~15% per month (figure 2).

### Potentially unsafe coprescribing of warfarin and a DOAC

**Figure 3:**
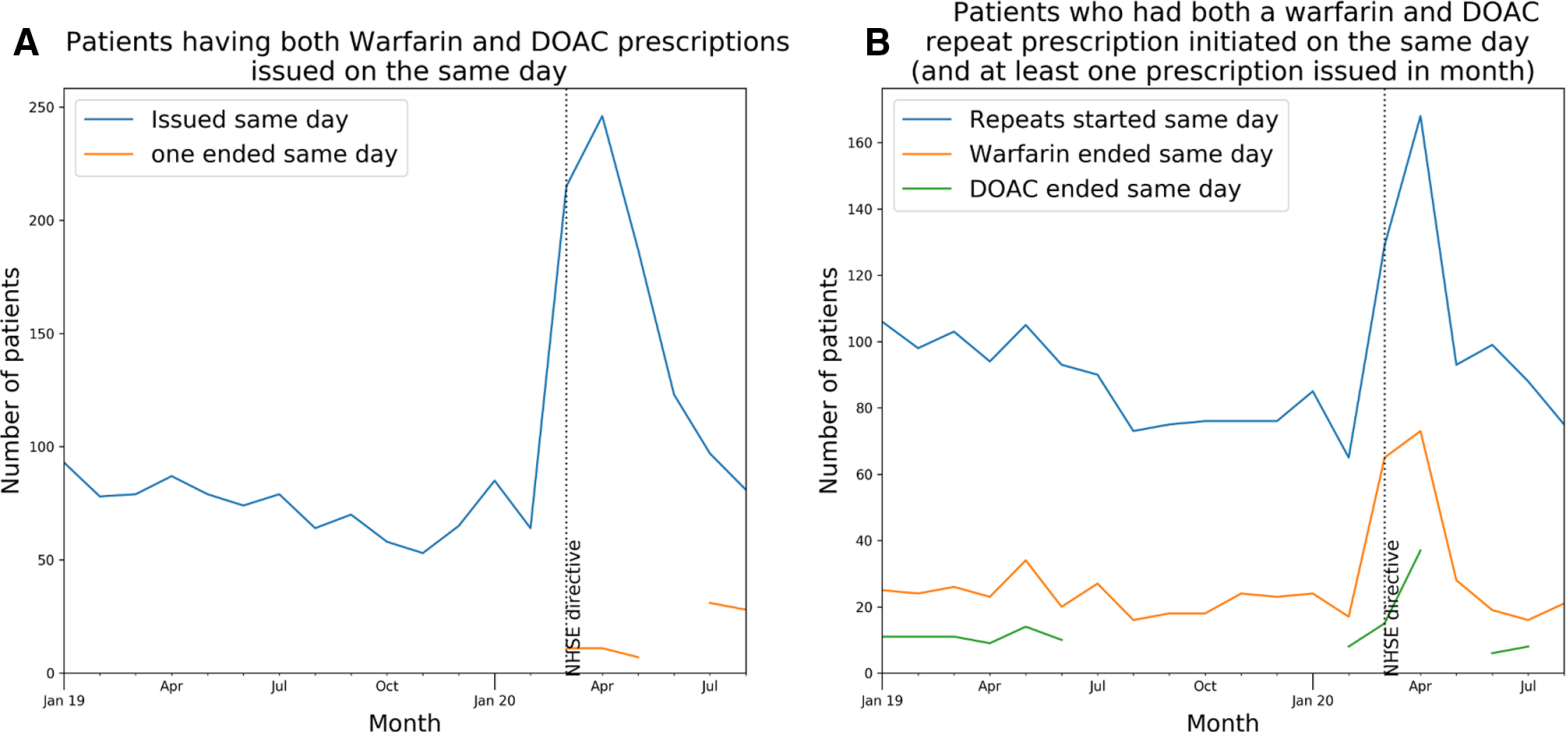
Patients coprescribed warfarin and a DOAC. Number of patients having (A) both warfarin and a DOAC prescription issued on the same day, also indicating how many patients for whom one of those prescriptions was cancelled the same day (ie, its end date was equal to its start date); (B) both warfarin and a DOAC repeat prescriptions initiated on the same day (restricted to patients who also had at least one warfarin or DOAC prescription issued in the given month), also indicating how many patients for whom one of those repeat prescriptions was cancelled the same day (ie, its end date was equal to its start date). Patient counts are shown in online supplemental table S2. DOAC, direct-acting oral anticoagulant.

Prior to the pandemic period, typically 50–100 people per month had both warfarin and a DOAC issued on the same day, rising sharply to 246 in April 2020 (0.06% of all people receiving a DOAC or warfarin) before declining gradually to almost baseline by August 2020 (figure 3A). Only a small proportion of these patients had at least one of their coprescriptions cancelled on the same day (ie, prescription end date equal to start date). Prior to the pandemic, 60–110 patients per month had both warfarin and a DOAC repeat prescription initiated on the same day (figure 3B). This figure reached a peak of ~170 in April and declined rapidly to near-normal levels from May 2020. However, this rise was compensated by an increase in the number of repeat prescriptions that were cancelled the same day (usually warfarin).

10.1136/openhrt-2021-001784.supp1 Supplementary data

### INR tests during the COVID-19 period

**Figure 4:**
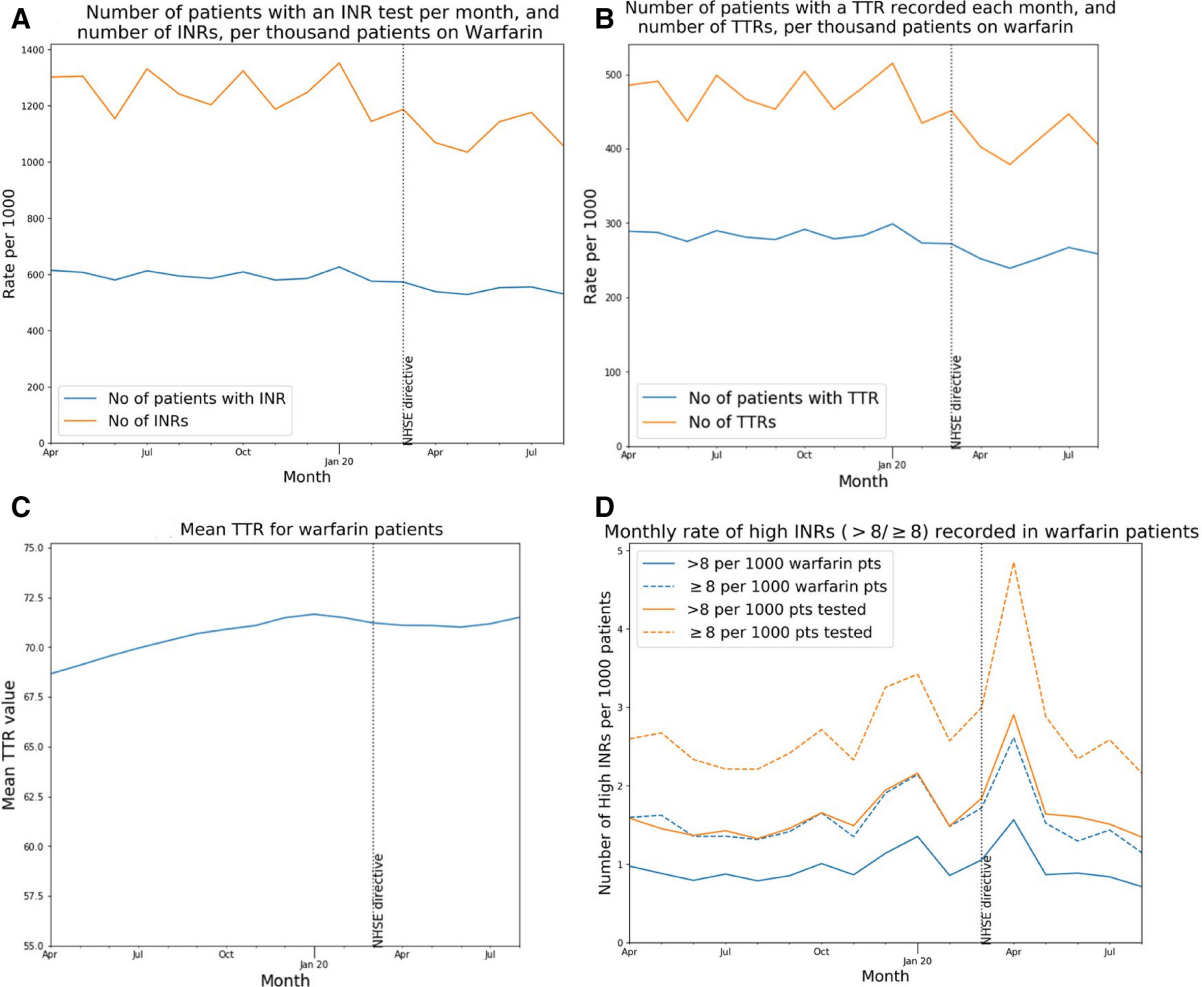
INR tests and recorded TTRs in warfarin patients. (A) Number of patients having an INR test, and the total number of INR tests carried out, per thousand warfarin patients. (B) Number of patients having a TTR recorded, and the total number of TTRs, per thousand warfarin patients. (C) Mean TTR value across warfarin patients tested. (D) Monthly rate of elevated INRs (as >8 and ≥8) recorded in warfarin patients per thousand warfarin patients and thousand patients having an INR test. INR, international normalised ratio; TTRs, time in therapeutic range.

Of 164 000 people on warfarin prior to March 2020, 80.1% had an INR test recorded and 35% had a TTR recorded between March and May 2020 (table 2). The number of warfarin patients tested each month was approximately constant prior to the pandemic (589 per 1000 eligible patients per month, January 2019–March 2020) but with a small reduction during the pandemic (figure 4A). The monthly testing rate reduced by 14%, to 506.8 (April–August 2020), a reduction of 82.4 per 1000 patients being tested per month (14.0% reduction). The number of TTRs recorded followed a similar pattern (figure 4B), and where TTR was recorded, the mean value for those who were still on warfarin remained relatively constant (figure 4C). Figure 4D, E illustrate the rate of high INRs per 1000 warfarin patients, per 1000 INR tests and per 1000 patients tested. A small peak is observed in April, but the absolute number of high INR results (≥8) was 470, only slightly higher than January’s figure of 420 (online supplemental table S1).

### Factors associated with switching from warfarin to a DOAC during the pandemic

A total of 149 243 people were included in this analysis (online supplemental table S3) and our logistic regression model results are presented in table 4. People who had reduced renal function were less likely to switch from warfarin to DOAC compared with those with eGFR ≥60 (eGFR <30: OR 0.51, 95% CI 0.46 to 0.57; eGFR 30–59: OR 0.92, 95% CI 0.88 to 0.96). Those with no eGFR in the last 12 months but who had had a renal function test recorded in the last 4 months were more likely to switch (OR 2.11, 95% CI 2.02 to 2.21), while those with no eGFR or recent renal function test were less likely to switch (0.40, 95% CI 0.38 to 0.42). People with multiple recent INR tests were more likely to switch (over seven vs none recorded, OR 1.88, 95% CI 1.77 to 1.99); older people (over 75 vs under 65, OR 1.89, 95% CI 1.77 to 1.99); people living in a care home (OR 1.32, 95% CI 1.15 to 1.51); and people with a diagnosis of AF (OR 1.95, 95% CI 1.87 to 2.03). People with a recorded contraindication for a DOAC were less likely to be switched (OR 0.37, 95% CI 0.26 to 0.52). We also found that Sustainability and Transformation Partnership (STP) membership, when modelled as a random effect, was a significant driver of variation (p<0.0001).

**Table 4:** Factors associated with switching from warfarin to a DOAC during the pandemic

		Univariable logistic regression		Mixed effects logistic regression*	
		OR	95% CI	OR	95% CI
Age (years)	Under 65	Ref		Ref	
	65–74	2.05	1.93 to 2.17	1.50	1.41 to 1.60
	75 and over	2.87	2.72 to 3.03	1.89	1.78 to 2.01
Index of multiple deprivation	Quintile 5 (least deprived)	Ref		Ref	
	Quintile 4	1.13	1.07 to 1.19	1.07	1.01 to 1.13
	Quintile 3	1.21	1.15 to 1.27	1.07	1.02 to 1.13
	Quintile 2	1.20	1.14 to 1.26	1.05	1.00 to 1.11
	Quintile 1 (most deprived)	1.20	1.14 to 1.27	1.03	0.97 to 1.08
Care home	No	Ref		Ref	
	Yes	1.47	1.29 to 1.68	1.32	1.15 to 1.51
Atrial fibrillation	No	Ref		Ref	
	Yes	2.49	2.39 to 2.58	1.95	1.87 to 2.03
eGFR	≥60	Ref		Ref	
	Not measured	0.38	0.36 to 0.40	0.40	0.38 to 0.42
	30–59	1.12	1.08 to 1.17	0.92	0.88 to 0.96
	<30	0.63	0.57 to 0.70	0.51	0.46 to 0.57
	Other RFT	2.20	2.10 to 2.30	2.11	2.02 to 2.21
Number of recent INR tests	0	Ref		Ref	
	1–3	1.50	1.42 to 1.58	1.35	1.28 to 1.42
	4–6	1.96	1.85 to 2.06	1.75	1.66 to 1.85
	7+	1.99	1.88 to 2.11	1.88	1.77 to 1.99
Length of warfarin prescription (years)	<2	Ref		Ref	
	2–≤6	1.69	1.54 to 1.85	1.26	1.14 to 1.38
	6–<8	1.89	1.72 to 2.07	1.31	1.19 to 1.44
	≥8	1.58	1.44 to 1.73	1.14	1.04 to 1.26
Previous DOAC prescription	No	Ref		Ref	
	Yes	0.65	0.60 to 0.70	0.65	0.60 to 0.70
DOAC contraindication	No	Ref		Ref	
	Yes	0.41	0.30 to 0.58	0.37	0.26 to 0.52

Patient counts are reported in online supplemental table S3.

*Adjusted for the variables in the table STP membership as a random effect.

DOAC, direct-acting oral anticoagulant.

## Discussion

### Summary

We observed a substantial increase in people switching from warfarin to a DOAC after NHS England advice to do so during the COVID-19 pandemic. We were able to identify in the data that a small but substantial number of people (n=246, 0.06%) simultaneously prescribed warfarin and a DOAC in April 2020 and were potentially at risk of serious adverse effects. Overall, the rate of INR testing for those on warfarin dropped by 14%, although we observed no substantial change in the proportion of INR tests that reported a clotting time outside the desired range. Factors associated with switching from warfarin to DOACs included older age, higher number of recent INR tests, diagnosis of AF, normal renal function and care home residency.

### Strengths and weaknesses

The key strength of this study is the scale, timeliness and completeness of the underlying data. The OpenSAFELY platform runs analyses across the full, raw, single-event-level medical records of all patients at 40% of all GP practices in England, including all tests, treatments, diagnostic events and other salient clinical and demographic information. We also recognise some limitations. We assessed the number of prescriptions issued or initiated on repeat prescriptions. We cannot currently access information on which medications were dispensed. An individual appearing to receive warfarin and a DOAC may therefore not receive, or take, both medications: for example, a doctor might notice the coprescribing some time after the consultation and cancel the prescription, or a pharmacist may decline to dispense both medications for safety reasons. Finally, a patient may be informed not to take the medicine by their healthcare professional even if they received a dispensed medicine. Nonetheless, this does not diminish the finding that coprescribing of warfarin and DOACs occurs and that incidence increased substantially during COVID-19 to over 200 people in 1 month. Another limitation relates to missing INR data: people in England often have their warfarin managed by an ‘anticoagulation clinic’ in a community service, hospital outpatient department or neighbouring general practice with specialist expertise; these clinics typically use bespoke software to record all relevant results related to INR, which may not be transferred in structured data into GP records but would be visible to clinicians delivering direct care for a patient; this could explain the apparent absence of INR tests for some people but not negatively impact clinical care. Our regression analysis could not capture all possible causes of confounding; in particular. the patient’s practice was excluded to allow the model to converge, and this would be expected to have a strong influence on anticoagulant prescribing.

### Findings in context

A recent systematic review of healthcare utilisation during the pandemic found that utilisation reduced by approximately one-third during the pandemic,^8^ but we are not aware of any published studies assessing switching of anticoagulant therapy during the pandemic or implementation of NHS England anticoagulation guidance. We found that edoxaban and apixaban were favoured DOACs when switching from warfarin, not wholly in line with NHS England advice from May 2020,^3^ which recommends apixaban and rivaroxaban. In October, NICE published draft guidance on AF recommending apixaban and dabigatran; however, we found dabigatran was prescribed in a very small proportion of switches.^9^ In England, general practitioners have autonomy in the selection of treatments they prescribe but are obliged to consider a wide number of factors that may influence choice of DOACs.^10^ Our previous research has shown NHS England guidance advising GPs to stop prescribing certain medicines that did not cause an immediate noticeable change in prescribing patterns,^11^ and CCG membership has a strong association with GP prescribing choices.^12–15^ Our study shows evidence of switching after the guidance and although we were not able to directly assess CCG membership, our study did find that STPs, an administrative region made up of one or more CCGs, were a significant driver of variation. The MHRA safety alert on anticoagulation identified a ‘small number of patients’ coprescribed warfarin and a DOAC but provided no further information on the scale of the problem. One Swiss hospital found 0.8% of its patients on anticoagulant therapy (121/15812) were coprescribed two anticoagulants in a single year.^16^ However, 88.7% of these cases involved coprescription of DOACs and low-molecular weight heparins, not included in our study. As regards INR tests, the MHRA reports appears to have been triggered by a root cause analysis from a single centre in London,^17^ reporting that between 1 March and 17 April 2020, 0.9% (30/3214) of INRs were high (>8.0) compared with 0.1% (6/4079) the previous year. Analysing the full records of 40% of patients in England, we found only a small peak in high INRs and with no obvious change in the mean TTR recorded for those still on warfarin.

### Policy implications for research and practice

The COVID-19 pandemic has brought new challenges for the NHS to deliver safe and effective routine care. NHS England issued anticoagulant guidance at the peak of the pandemic and a substantial number of people were switched in line with this guidance. This study using OpenSAFELY demonstrates it is possible to use routinely collected raw EHR data on a specific clinical area to support evaluation of national guidance and safety alerts during the COVID-19 pandemic. There is a need for high quality applied practical research to support healthcare organisations’ responses to the pandemic; OpenSAFELY can be used by NHS England, the MHRA and others such as NICE to rapidly assess in near-real time the impact of policy and clinical guidance as well as informing future versions of guidance. Specific areas of focus for anticoagulants during COVID-19 could include: identifying people eligible for switching who have not switched yet; assessing the scale of clinical work needed; evaluating completeness of uptake; prioritising risk groups; understanding causes of coprescription of warfarin and DOACs; evaluating variation in organisations response to new guidance^14^; and better understanding the factors identified by MHRA as being associated with elevated INRs such as coprescribing of antibiotics.^4^

### Summary

We observed increased switching of anticoagulants from warfarin to DOACs at the outset of the COVID-19 pandemic in England. We did not find a widespread rise in elevated INR test results that may be reassuring after a recent MHRA safety alert, although we did observe a small but substantial number of people who were coprescribed warfarin and DOACs.

## Data Availability

Data are available in a public, open access repository. Data management was performed using Python 3.8 and SQL, and regression analysis using Stata 16.1. All code for the OpenSAFELY platform, and for data management and analyses for this study, are available for inspection and reuse under open licenses on GitHub (https://github.com/opensafely/anticoagulant-switching-research). All codelists are available for inspection and re-use from https://codelists.opensafely.org/.
