# Supplementary material for: OpenSAFELY: impact of national guidance on switching anticoagulant therapy during COVID-19 pandemic
Source: Open Heart. 2021 Nov 16;8(2):e001784. doi: 10.1136/openhrt-2021-001784 (PMC8595296; doi:10.1136/openhrt-2021-001784)
Supplement: Supplementary data [file openhrt-2021-001784supp001.pdf]

OpenSAFELY: impact of national guidance on switching anticoagulant therapy during COVID-19 pandemic

Supplementary material

Table S1: *Patient count and test count for high INR results per month of 2020, rounded to the nearest 10.*

		8 exactly	over 8	Total (≥8)
Patient count	Jan 2020	160	260	**420**
	Feb 2020	120	160	**280**
	Mar 2020	120	200	**320**
	Apr 2020	190	280	**470**
	May 2020	110	150	**260**
	Jun 2020	70	140	**210**
	Jul 2020	90	130	**220**
	Aug 2020	60	110	**170**
	Sep 2020	80	140	**220**
Test count	Jan 2020	200	310	**510**
	Feb 2020	160	190	**350**
	Mar 2020	160	240	**400**
	Apr 2020	250	340	**590**
	May 2020	130	180	**310**
	Jun 2020	90	180	**270**
	Jul 2020	110	160	**270**
	Aug 2020	80	120	**200**
	Sep 2020	100	170	**270**

Table S2: *Patients issued warfarin and DOAC scripts on the same day. Values below 5 are shown as "1-5".*

Month	Warfarin and DOAC issued same day	One ended same day
Jan 2019	93	1-5
Feb 2019	78	1-5
Mar 2019	78	1-5
Apr 2019	87	1-5
May 2019	79	1-5
Jun 2019	74	1-5
Jul 2019	79	1-5
Aug 2019	64	1-5
Sep 2019	70	1-5
Oct 2019	58	0
Nov 2019	52	1-5
Dec 2019	65	1-5
Jan 2020	85	1-5
Feb 2020	64	1-5
**Mar 2020**	**214**	10
**Apr 2020**	**246**	11
May 2020	187	7
Jun 2020	122	1-5
Jul 2020	95	31
Aug 2020	81	28

Table S3: *Number of patients included in regression for factors associated with switching from warfarin to a DOAC during the pandemic, stratified by the outcome of whether the patient was switched to a DOAC.*

**Factor**		**DOAC switched?**	
		No	Yes
All patients	*N=149,243*	129,336	19,907
Age	Under 65	25,327	1,731
	65 to 74	33,291	4,579
	75 and over	70,718	13,597
Ethnicity	Unknown	37,086	5,733
	White	88,751	13,871
	Mixed	309	25
	South Asian	1,910	159
	Black	733	67
	Other	547	52
IMD	Least deprived	26,610	3,331
	|	25,934	3,846
	|	25,920	4,390
	|	25,238	4,132
	Most deprived	25,634	4,208
Care home	No	127,993	19,621
	Yes	1,343	286
Atrial fibrillation	No	44,792	3,577
	Yes	84,544	16,330
eGFR	>=60	43,872	2,830
	Not measured	44,491	7,562
	30-59	26,748	5,071
	<30	3,965	435
	Other recent RFT	10,260	4,009
Number of recent INR tests	0	23,552	2,303
	1-3	53,250	7,631
	4-6	30,412	5,785
	7+	22,122	4,188

Length of warfarin prescription	<= 2 years	6,066	572
	2-6 years	30,917	4,847
	6-8 years	24,464	4,322
	>8 years	67,889	10,166
Previous DOAC prescription	No	122,406	19,148
	Yes	6,930	759
DOAC contraindication	No	128,804	19,870
	Yes	532	37
